# The effect of vitamin D supplementation on survival in patients with colorectal cancer: systematic review and meta-analysis of randomised controlled trials

**DOI:** 10.1038/s41416-020-01060-8

**Published:** 2020-09-15

**Authors:** Peter G. Vaughan-Shaw, Louis F. Buijs, James P. Blackmur, Evi Theodoratou, Lina Zgaga, Farhat V. N. Din, Susan M. Farrington, Malcolm G. Dunlop

**Affiliations:** 1 MRC Human Genetics Unit, Institute of Genetics and Molecular Medicine, University of Edinburgh, Edinburgh, UK; 2 Cancer Research UK Edinburgh Centre, Institute of Genetics and Molecular Medicine, University of Edinburgh, Edinburgh, UK; 3 Centre for Global Health Research, Usher Institute for Population Health Sciences and Informatics, University of Edinburgh, Edinburgh, UK; 4 Department of Public Health and Primary Care, Trinity College Dublin, Dublin, Ireland

**Keywords:** Outcomes research, Chemotherapy, Colon cancer, Rectal cancer, Cancer epidemiology

## Abstract

**Background:**

Low circulating vitamin D levels are associated with poor colorectal cancer (CRC) survival. We assess whether vitamin D supplementation improves CRC survival outcomes.

**Methods:**

PubMed and Web of Science were searched. Randomised controlled trial (RCTs) of vitamin D supplementation reporting CRC mortality were included. RCTs with high risk of bias were excluded from analysis. Random-effects meta-analysis models calculated estimates of survival benefit with supplementation. The review is registered on PROSPERO, registration number: CRD42020173397.

**Results:**

Seven RCTs (*n* = 957 CRC cases) were identified: three trials included patients with CRC at outset, and four population trials reported survival in incident cases. Two RCTs were excluded from meta-analysis (high risk of bias; no hazard ratio (HR)). While trials varied in inclusion criteria, intervention dose and outcomes, meta-analysis found a 30% reduction in adverse CRC outcomes with supplementation (*n* = 815, HR = 0.70; 95% confidence interval (CI): 0.48–0.93). A beneficial effect was seen in trials of CRC patients (progression-free survival, HR = 0.65; 95% CI: 0.36–0.94), with suggestive effect in incident CRC cases from population trials (CRC-specific survival, HR = 0.76; 95% CI: 0.39–1.13). No heterogeneity or publication bias was noted.

**Conclusions:**

Meta-analysis demonstrates a clinically meaningful benefit of vitamin D supplementation on CRC survival outcomes. Further well-designed, adequately powered RCTs are needed to fully evaluate benefit of supplementation in augmenting ‘real-life’ follow-up and adjuvant chemotherapy regimens, as well as determining optimal dosing.

## Background

Colorectal cancer (CRC) is the third most common cancer across the world, with 1.8 million cases and ~860,000 deaths each year.^1^ There is a 10-fold variation in incidence across the world with risk being highest in developed countries, suggesting that the disease may be largely preventable. Ecological variation in vitamin D levels between populations has been proposed as an environmental factor contributing to variation in CRC incidence.^2^

Controversy surrounds the role of vitamin D deficiency in the aetiology of several common cancers. The strongest available observational evidence supports a link between vitamin D and CRC.^3–5^ Numerous in vitro studies demonstrate vitamin D-induced growth arrest and apoptosis of CRC cells, modulation of the *Wnt* signalling pathway, DNA repair and immunomodulation,^6^ lending support to a causal relationship between vitamin D and cancer. However, observational data implicating vitamin D deficiency in CRC aetiology or survival are limited by potential bias: environmental risk factors associated with CRC are also associated with vitamin D status (co-causality; e.g. physical activity, obesity); heterogeneity in assay type and performance across studies; the development of CRC itself—or its treatment—may induce lower vitamin D levels (reverse causation).^5^ Mendelian randomisation is an approach that can provide evidence for causality, but studies have thus far failed to detect a causal association between blood 25-hydroxyvitamin D level and CRC risk.^7^ This may be due to weakness of the available genetic instruments, combined with powerful environmental influences, such as variation in exposure to vitamin D-making ultraviolet B (UVB) sunlight.

Large population trials to date, including the VITAL, VIDA and WHI trials, have shown that vitamin D supplementation did not provide any detectable difference in the *incidence* of CRC.^8–10^ Baron et al.^11^ also reported no reduction in risk of recurrent colorectal adenomas following 3–5 years of supplementation. However, several features of these studies may have limited the ability to detect any effect of supplementation on clinical endpoints.^12,13^ In brief, recruited subjects were predominantly already sufficient or replete for vitamin D, thereby blunting any health benefit that might be achieved; ‘off-protocol’ vitamin D supplementation was reported in control groups; population heterogeneity such as genetic (variable response or action of vitamin D due to participant genetics) and UVB exposure due to latitude of residence/outdoor activity was not considered as CRC incidence rate was low during follow-up.

In support of a causal effect, several studies have demonstrated an interaction between vitamin D-related genetic variation, 25-hydroxyvitamin D (25OHD) level and CRC or neoplasia risk or outcome, mitigating against potential confounding effects.^14–17^ In a sub-analysis of VITAL trial data, a lower rate of all cancer death was observed after 2 years of follow-up (hazard ratio (HR) = 0.75; 95% confidence interval (CI 0.59–0.96)). Furthermore, a recent meta-analysis found reduced total cancer mortality with vitamin D supplementation (HR = 0.87; 95% CI 0.79–0.96).^18^

Here, we present a systematic review and meta-analysis of randomised controlled trials examining the impact of vitamin D supplementation on progression and survival in patients with CRC.

## Methods

### Literature search

We performed two literature searches. First, to identify trials of vitamin D supplementation in CRC patients; second, to identify completed trials of vitamin D supplementation in non-cancer cohorts, which included cancer mortality as a trial outcome. The electronic databases PubMed^19^ and Web of Science^20^ were systematically searched for eligible trials from inception until 31 January 2020.

A comprehensive list of search terms directly relevant to the scope of this systematic review was created. For vitamin D, we included a wide range of terms, including vitamin D, 25-hydroxyvitamin D, calcidiol, cholecalciferol and 25OHD. For the intervention, the following terms were used: supplementation, intervention, treatment, placebo and RCT. For the patient population, we included terms: CRC, bowel, digestive, colon and rectum. Last, for the outcome we included terms: survival, prognosis, mortality and recurrence (Supplementary Table 1). For trials in non-cancer cohorts, the CRC terms were omitted (i.e. CRC, bowel, digestive, colon and rectum). We considered all human research original full-text articles with no restriction on follow-up duration or language, but excluded case reports, reviews and prior meta-analyses. The two searches returned 768 and 3333 articles, respectively. Bibliographies from obtained articles, relevant reviews and clinicaltrials.gov were searched with no further relevant and reported trials identified. To ensure all relevant trials had been included, we checked results against two recent meta-analyses of vitamin D supplementation and all cancer mortality,^18,21^ which did not yield any further trials. Titles/abstracts were screened by two researchers (P.G.V.-S. and L.F.B.), who then screened full texts for eligibility. The trial ‘PICO’ inclusion criteria were: (i) participants: individuals over the age of 18 years (with/without diagnosis of CRC); (ii) intervention: vitamin D supplementation; (iii) comparators: a placebo/lower dose of vitamin D; (iv) outcome: all measures of survival, for example, progression-free survival, overall survival (OS) and CRC-specific survival. Only randomised controlled trials were included. Disagreements at any stage were resolved by discussion with the senior author (M.G.D.). The review is registered on PROSPERO, registration number CRD42020173397.

### Data extraction

Data extraction was conducted by two investigators (L.F.B. and P.G.V.-S.). The data from eligible trials were extracted into a prospectively designed database, including the following information: trial name, publication year, location, sample size, the trial duration, the active intervention (dose and frequency) and comparator (placebo or lower dose), treatment duration and total follow-up duration, the primary and secondary outcomes and the measured outcome (e.g. HR for OS, disease-free survival (DFS) or relapse-free survival and colorectal/disease-specific survival (DSS)). The most fully adjusted HR were extracted. Where the relevant HR were not reported, we contacted the trial authors by email to obtain these (*N* = 4 contacted, two authors provided relevant HR). For population trials, we included HR for CRC mortality from the time of randomisation in those trial subjects who developed CRC.

### Quality assessment

An assessment of the methodological quality of the included trials was conducted using the 2010 CONSORT statement by two authors (L.F.B. and P.G.V.-S.) and disagreement resolved by discussion. Each trial was assessed for adherence against the CONSORT checklist as per previously reported methods.^22–24^ Adherence against 22 items was assessed and any trial with a high level of missing items (>50%) was considered at high risk of bias and excluded from quantitative assessment through meta-analysis.

### Statistical analysis

The main analysis was a trial level meta-analysis of supplementation and CRC outcomes for all eligible trials. Secondary pre-specified sub-group meta-analyses were individually performed for colorectal-specific survival and DFS and for CRC and population trials. The extracted HRs and 95% CIs were used to calculate the pooled HR estimates. Standard errors were used to calculate weighting for each trial. The Hartung–Knapp–Sidik–Jonkman method was used to calculate pooled HR because of the a priori expected heterogeneity between trials, due to differences among populations and methodological dissimilarities between trials. This method was preferred over the DerSimonian and Laird random-effects model given the small number of trials included in the meta-analysis.^25,26^ The *I*^2^ statistic was calculated to quantify the degree of heterogeneity between trials and assess impact on the meta-analysis.^27^ Publication and selection bias was investigated by checking for asymmetry in the funnel plots and running the Egger’s regression test.^28^ All analyses were performed in R^29^ with the R-package ‘metafor’ used for meta-analyses.^30^

## Results

### Literature search

**Fig. 1:**
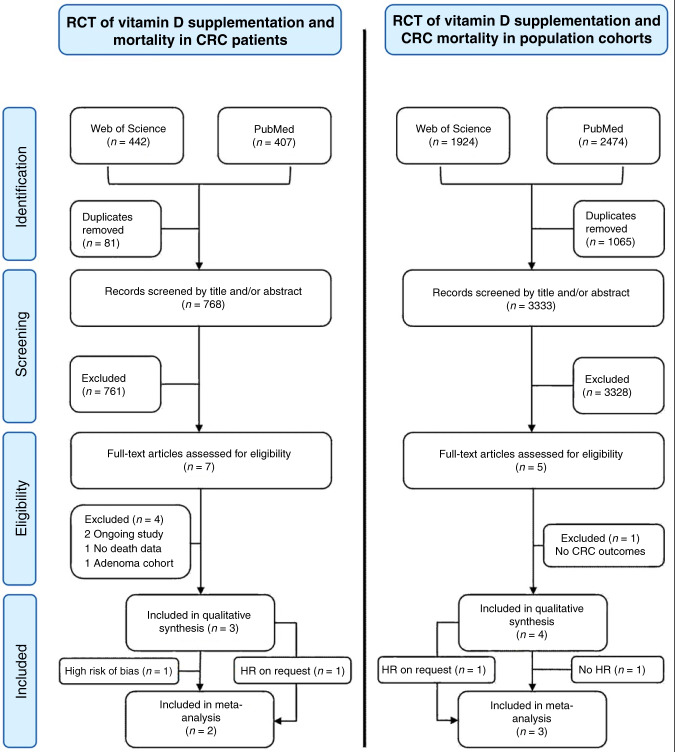
PRISMA flowchart of the trial selection process. Excluded RCTs of supplementation in CRC patients were the D-health trial (ongoing trial,^40^) D2dca trial (not yet published); trials by Lappe et al.^52^ solely reported cancer incidence, but not survival outcomes; Baron et al.^11^ reported CRC incidence in an adenoma cohort, but with only 14 CRC cases.^53^ The excluded RCT of supplementation in population cohorts was the VIDA trial as CRC deaths were not reported or available on request;^10^ the RECORD trial reported CRC deaths,^39^ but a hazard ratio (HR) was not reported or available on request, so was excluded from meta-analysis. The Golubic et al.^31^ trial was not included in the meta-analysis of trials in CRC patients due to a high risk of bias, see below.

A flowchart illustrating trial selection process is shown in Fig. 1. After removal of duplicates, the two searches (in CRC patients and population trials) yielded 768 and 3333 trials, respectively. Full texts of seven trials in CRC patients and five population trials were considered for inclusion and assessed for eligibility. Full-text review and subsequent correspondence with trial authors yielded three relevant trials in CRC patients^31–33^ and four population trials for systematic review^8,34,35^ (Table 1).

**Table 1:** Characteristics of included trials.

Name (country, year)	Rx duration	Total/CRC cases^a^	Participant population	Intervention	Comparator	Primary outcome	Relevant secondary outcome	Follow-up from Rx start	CRC deaths	Overall survival	CRC-specific survival	Progression-free survival
CRC/adenoma trials												
Golubic et al.^31^ (Croatia, 2018)	2 years	71/71	Metastatic CRC, age 24–79 years	2000 IU/day vitamin D_3_ + standard chemotherapy	No placebo	OS	PFS	46 months	Not known	HR = 1.01 (95% CI 0.39–2.61)	NA	HR = 1.11 (95% CI 0.69–1.77)
SUNSHINE^32^ (USA, 2019)	23 months	139/139	Metastatic CRC	4000 IU/day vitamin D3 + standard chemotherapy	400 IU/day vitamin D_3_	PFS	OS	23 months	111	24.3 vs. 24.3 months; *P* = 0.43	NA	HR = 0.64 (95% CI 0–0.90)
AMATERASU^33^ (Japan, 2019)	3.5 years	201/201	Epithelial carcinoma of digestive tract (stages 1–3)	2000 IU/day vitamin D_3_ + standard chemotherapy	Placebo	PFS	OS	3.5 years	Not known	HR = 0.95 (95% CI 0.57–1.57)	NA	HR = 0.69 (95% CI 0.39–1.24)
Population trials												
Trivedi et al.^34^ (UK, 2003)	5 years	2686/55	Aged 65–85 years	100,000 IU/4m vitamin D_3_	Placebo	Fracture incidence, mortality	Colon-DSS	NA	18	NA	HR = 0.62 (95% CI 0.24–1.60)	NA
WHI trial^7^ (USA, 2006)	7 years	36,282/322	Post-menopausal women, 50–79 years	400 IU/day vitamin D_3_ + CaCO_3_	Placebo	Hip fracture	CRC risk	7 years	75	HR = 0.91 (95% CI 0.83–1.01); (total trial mortality)	HR0.82 (95% CI 0.52–1.29)	NA
RECORD trial^39^ (UK, 2012)	24–62 months	5292/71	Aged >70 years, fracture	800 IU/day vitamin D3 ± calcium	Placebo ± calcium	Fracture	OS, PFS	24–62 months	33	HR = 0.80 (95% CI 0.61–1.11) (total trial mortality)	20/41 deaths (vitamin D3) vs.13/30; *P* = 0.83^†^	NA
VITAL trial^35^ (USA, 2019)	5 years	25,871/98	Men ≥50 years, women ≥55 years	2000 IU/day vitamin D_3_ + omega-3 fatty acids	Placebo	Invasive cancer risk	Cancer mortality	5.3 years	25	NA	HR = 0.65 (95% CI 0.28–1.50)	HR = 0.79 (95% CI 0.36–1.75)

*Rx* intervention/comparator duration, *IU* International units, *4m* every 4 months, *CaCO*_*3*_ calcium carbonate, *OS* overall survival, *PFS* progression-free survival, *DSS* disease (CRC)-specific survival, *colon-DSS* colon cancer disease-specific survival, *HR* hazard ratio with 95% CI given within parentheses, *NA* not available.

^a^CRC number given as recruited cases or incident cases.

^†^*P* value was not reported in this paper; *χ*^2^ test was performed here.

The main characteristics of included trials are summarised in Table 1. In brief, Golubic et al.^31^ found no effect of supplementation on OS in stage IV patients at 46 months (2000 IU/day; baseline median 25OHD 13.2 ng/ml, 70 (98.6%) of 71 patients insufficient (<20 ng/ml) at baseline, survival HR = 1.01; 95% CI: 0.39–2.61). In the SUNSHINE trial,^32^ 70% (*n* = 87) patients had insufficient 25OHD at baseline, and 4000 IU/day supplementation increased median 25OHD from 16.1 to 34.8 ng/ml (87 (70%) with improved median progression-free survival from 11.0 to 13.0 months in stage IV CRC patients (HR = 0.64; 95% CI: 0–0.90; median follow-up 23 months). In the AMATERASU trial,^33^ 41% (*n* = 173) patients had insufficient 25OHD at baseline, with 2000 IU/day supplementation associated with a non-significant improvement in survival with supplementation after median follow-up 3.5 years in stage I–III patients (25OHD ~20 ng/ml at baseline, ~60 ng/ml at follow-up; HR = 0.69; 95% CI: 0.39–1.24). In the population trials, 400 IU/day in the Women’s Health Initiative trial^8^ did not impact CRC mortality (baseline median 25OHD 18.4 ng/ml; proportion of insufficient participants at baseline not given; HR = 0.82; 95% CI: 0.52–1.29), with similar results reported by Trivedi et al.^34^ (100,000 IU/4-monthly; proportion of insufficient participants at baseline not given; follow-up 25OHD 29.7 vs. 21.4 ng/ml with placebo; HR = 0.62; 95% CI: 0.24–1.60). The VITAL trial^35^ authors provided relevant data on request, with a trend towards increased DSS and PFS in 98 incident CRC cases (only 2001 (13%) insufficient for 25OHD at baseline; 25OHD 29.8 ng/ml to 41.8 ng/ml in treatment arm; DSS HR = 0.65; 95% CI: 0.28–1.50); PFS HR = 0.79; 95% CI: 0.36–1.75). In the RECORD trial^36^ of secondary fracture prevention, there was no impact on CRC death in 71 incident CRC cases, with 20 CRC deaths in the vitamin D group and 13 in the placebo/calcium groups (baseline 25OHD 15.2 ng/ml; HR not available on request).

### Quality assessment

Adherence to the CONSORT 2010 checklist^37^ was assessed for the seven trials identified in the literature search, with high rates of adherence for all but the Golubic trial (Supplementary Table 2). In particular, it was noted that this trial was not placebo controlled, with no reported mechanism to implement the random allocation sequence, no eligibility criteria for participants given and no description of level and method of blinding given. As a result, this trial was considered at high risk of bias and excluded from overall meta-analysis.

### Meta-analysis of vitamin D supplementation and survival outcomes

**Fig. 2:**
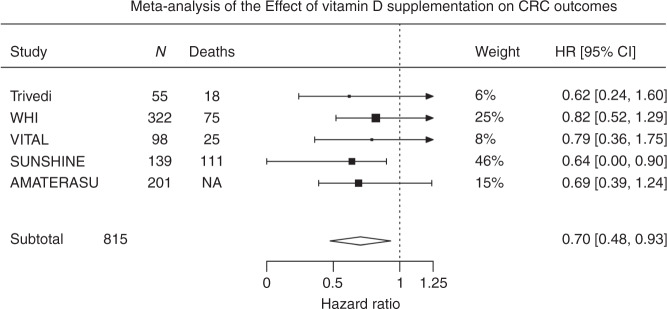
Meta-analysis of the effect of vitamin D supplementation on CRC outcomes. HRs used are for disease (CRC)-specific survival for the Trivedi and Women’s Health initiative (WHI) trials and progression-free survival for the VITAL, SUNSHINE and AMATERASU trials. HR confidence interval in the SUNSINE trial was one-sided. There was no evidence of heterogeneity with *τ*: 0.026; *I*^2^ (total heterogeneity/total variability): 0.85% and *P* = 0.98.

**Fig. 3:**
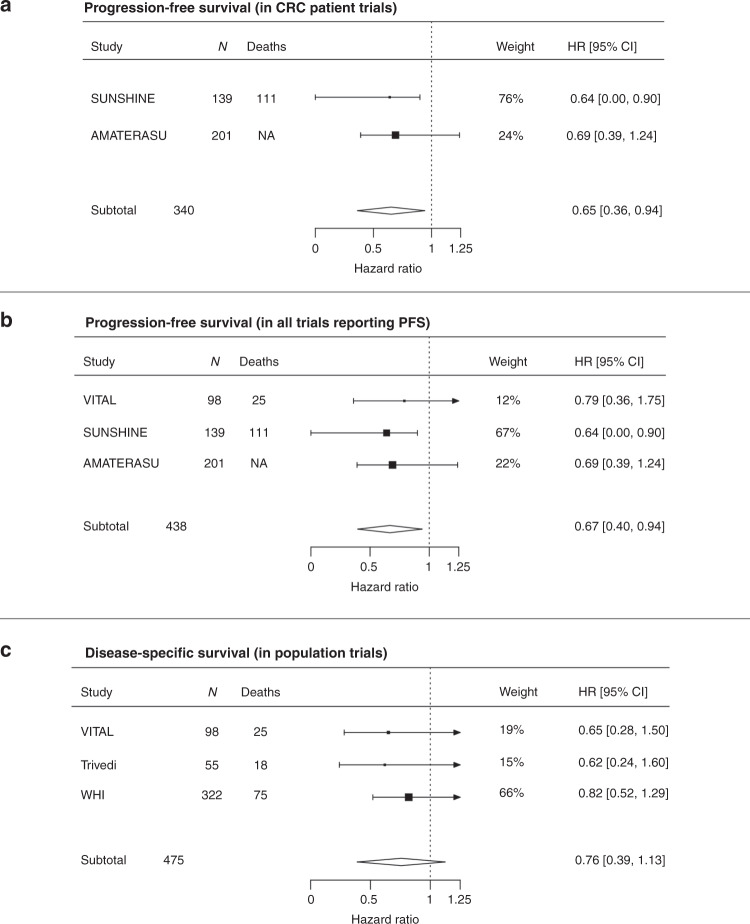
Sub-group meta-analyses results. HRs used are for disease (CRC) specific survival for the Trivedi and Women’s Health initiative (WHI) trials and progression-free survival for the VITAL, SUNSHINE and AMATERASU trials. Statistical testing in the SUNSINE trial was one-sided. There was no evidence of heterogeneity in sub-group meta-analyses presented in **a**–**c** with *τ* < 0.03; *I*^2^ (total heterogeneity/total variability): <0.6% and *P* > 0.88.

All included trials demonstrated a beneficial effect. Overall meta-analysis in five trials, comprising 815 participants revealed a beneficial effect of vitamin D supplementation on cancer outcomes in patients with CRC (HR = 0.70; 95% CI: 0.48–0.93; Fig. 2). Sub-group meta-analyses demonstrated a consistent favourable effect with vitamin D supplementation. In trials recruiting patients with CRC at outset, CRC progression or death was reduced by 35% (HR = 0.65; 95% CI:0.36–0.94; Fig. 3a), and by 33% across the three trials reporting PFS (HR = 0.67; 95% CI: 0.40–0.94; Fig. 3b). In the population trials, disease-specific survival improved by 24% (HR = 0.76; 95% CI: 0.39–1.13; Fig. 3c). Results were not quantitatively changed when the excluded Golubic et al.^31^ trial was included in the meta-analysis (Supplementary Fig. 1).

### Testing for trial heterogeneity and publication bias

Despite the different interventions and outcomes in the included trials, there was no evidence of heterogeneity with *τ*: 0.026 and *I*^2^: 0.85% in the overall meta-analysis. No evidence of publication bias was seen, with Egger’s regression test for funnel plot asymmetry *P* = 0.87 (Supplementary Fig. 2).^38^

## Discussion

This is the first systematic review with meta-analysis of randomised controlled trials to examine the effect of vitamin D supplementation on survival outcomes in patients with CRC. We found that supplementation imparts a 30% reduction in adverse survival outcomes overall, with a 24% reduction in CRC-specific death and a 33% in disease progression or death. The effect on survival was consistently observed in sub-group analyses both in trials specifically including CRC patients and in population trials reporting outcomes in incident CRC cases.

We included two RCTs of supplementation in patients with a diagnosis of CRC and demonstrated a 35% reduction in CRC progression or death with supplementation. We also recognised that incident cases of CRC occur in large population trials, providing an additional source of trial evidence. We included three population trials totalling almost 65,000 participants in our meta-analysis, with a suggestive benefit from supplementation on CRC-specific survival (HR = 0.76; 95% CI: 0.39–1.12). Two relevant trials were not included as HRs for CRC outcomes were not available after requests to the author,^10,39^ while we identified several ongoing trials yet to publish results, or example, the D-Health trial.^40^

The VITAL trial authors recently performed a review and meta-analysis of supplementation and all cancer mortality based on incident cancers in population supplementation trials,^18,35^ reporting a reduction in total cancer mortality with supplementation (HR = 0.83; 95% CI: 0.67–1.02). A similar meta-analysis by Zhang et al.^21^ found a similar effect (HR = 0.84; 95% CI: 0.74–0.95), yet combining all cancers may be flawed given that ‘cancer’ is a not a single disease, but a hugely heterogeneous group of individual and specific diseases. The current literature review is the first to assimilate evidence from trials specifically including patients with a diagnosis of CRC, but also large population trials that reported survival outcomes in incident CRC cases. A consistent reduction in adverse survival outcomes irrespective of the trial inclusion criteria, supplementation dose or survival outcome measure is supportive of a true causal effect, which supports observational data linking 25OHD level and cancer outcomes.^16,17^

There are a number of limitations in the currently available trial data impacting on this analysis. First, our literature search demonstrates a lack of well-designed and adequately powered randomised controlled trials investigating vitamin D supplementation and CRC outcomes. All included trials in the current meta-analysis were small, each including <500 CRC cases amounting to only 815 cases in meta-analysis. Next, the population trials included here did not report any data on stage, site or subtype of incident CRC cases or adjuvant therapy used, which are known to impact survival outcomes and the variables used for the HR adjustment are not consistently reported. Third, observational data strongly supports an association between genetic factors related to vitamin D metabolism or function and survival outcomes,^14,16,17^ yet no trial to date has considered the relevance of genetic heterogeneity to the impact of vitamin D on cancer death. Finally, we acknowledge that pooling estimates from trials with differing methodology may limit the conclusions that can be drawn. For example, in the population trials, the two groups are comparable at point of randomisation, but may not be comparable at point of diagnosis of CRC, which could bias outcomes. However, variability in inclusion criteria, interventions or outcomes generally results in a more heterogeneous estimate and is likely to increase statistical uncertainty and hence results tend towards the null. Nonetheless, our summary findings (i.e. direction and magnitude of effect size) remain largely unchanged when the analysis was limited according to trial methodology or outcome.

We acknowledge that translation of results from supplement RCTs to a real-life healthcare setting is not always straight forward. While vitamin D is cheap and generally safe, vitamin D intoxication or other adverse effects of supplementation must be considered. Poor compliance may also impact on real-life benefit. Lower 25OHD level is strongly associated with CRC survival in observational data,^14,16,17^ providing a strong rationale for supplementation trials in cancer patients with survival outcomes as the defined endpoint yet observational studies of vitamin D supplementation or intake and survival do not provide consistent evidence of benefit from vitamin D. A Norwegian study recently reported better CRC survival in incident CRC cases with pre-diagnostic vitamin D intake of >400 IU/day (HR = 0.75; 95% CI: 0.61–0.92).^41^ Similarly, the Cancer Prevention Study-II reported a trend towards greater OS in those with higher total or dietary vitamin D intake (HR = 0.88; 95% CI: 0.57–1.35 and HR = 0.90; 95% CI: 0.67–1.21), yet even in quartile four, the intake was low (~>245 IU/day).^42^ Jeffreys et al.^43^ reported a non-significant reduction in mortality after CRC diagnosis in women who had been prescribed vitamin D supplementation in the 5 years preceding CRC diagnosis (13% of 4122 cases prescribed supplements; HR = 0.90; 95% CI: 0.78–1.04), yet some other studies have found no benefit from low-dose supplementation.^44–46^ Crucially, all of these studies assess low doses of supplementation or intake and do not consider vitamin D-related genetic variants that have been shown to influence the association between vitamin D and survival.^14,16,17^ The lack of consistent findings in the observational data support further well-powered trials investigating the role of appropriate supplementary doses of vital D in CRC patients with insufficient 25OHD levels at baseline. The above findings, together with the clear benefit of 4000 IU over 400 IU in the SUNSHINE study, suggest that an intake of 400 IU/day is inadequate. Indeed, it is noted that the reference nutrient intake for vitamin D of 400 IU/day is recommended for the UK population, with this intake given as the average amount needed by 97.5% of the population to maintain a serum 25OHD concentration ≥10 ng/l when UVB sunshine exposure is minimal.^5^ The optimal dose for survival benefit remains unclear and requires further investigation, but given that data from several publications and national bodies indicates 2000–4000 IU/day to be safe,^5,47–51^ we believe that doses of ~2000–4000 IU should be considered for future trials.

In conclusion, this meta-analysis demonstrates a clinically meaningful beneficial effect from vitamin D supplementation on survival outcomes in patients with CRC. Further well-designed, adequately powered RCTs are needed to fully evaluate the benefit of supplementation in augmenting ‘real-world’ follow-up and adjuvant chemotherapy regimens, as well as determining optimal dosing.

## Supplementary information

Supplementary information

## Data Availability

Available at reasonable requests from pvaughan@ed.ac.uk.
