# Supplementary material for: The effect of vitamin D supplementation on survival in patients with colorectal cancer: systematic review and meta-analysis of randomised controlled trials
Source: Br J Cancer. 2020 Sep 15;123(11):1705–12. doi: 10.1038/s41416-020-01060-8 (PMC7686489; doi:10.1038/s41416-020-01060-8)
Supplement: Supplementary file 1 — Supplementary information [file 41416_2020_1060_MOESM1_ESM.docx]

**Supplementary Table 1 Search terms used for literature search**

|  | **Terms** | |
| --- | --- | --- |
| **Theme** | **Trials in Colorectal Cancer Patients** | **Population Trials reporting Colorectal Cancer Outcomes** |
| **Vitamin D** | (vitamin D) OR (25-hydroxyvitamin D) OR (25 hydroxyvitamin D) OR (25-hydroxy vitamin D) OR (25 hydroxy vitamin D) OR (calcidiol) OR (cholecalciferol) OR (25OHD) OR (25OH(D)) OR (25(OH)D) | |
| **Intervention** | (supplement*) OR (intervention) OR (treatment) OR (RCT) OR (randomis *) | |
| **Population** | ((cancer) OR (neoplasm) OR (malignant) OR (malignancy)) **AND**  ((colorectal) OR (bowel) OR (digestive) OR (colon) OR (rectal) OR (rectum) OR (intestine) OR (CRC)) | (cancer) OR (neoplasm) OR (malignant) OR (malignancy) |
| **Outcome** | (survival) OR (outcome) OR (prognosis) OR (mortality) OR (death) | |

**Supplementary Table 2 Adherence to CONSORT checklist**

| **First Author** | **Title & Abstract** | **Introduction** | **Methods** | **Results** | **Discussion** | **Total†** |
| --- | --- | --- | --- | --- | --- | --- |
| **Maximum** | **1** | **1** | **10** | **7** | **3** | **22** |
| Golubic *et al.* | 0 | 1 | 3 | 4 | 1 | **9** |
| SUNSHINE | 1 | 1 | 10 | 7 | 3 | **22** |
| AMATERASU | 1 | 1 | 10 | 7 | 2 | **21** |
| Trivedi *et al.* | 1 | 1 | 7 | 7 | 2 | **18** |
| WHI | 1 | 1 | 8 | 3 | 3 | **16** |
| RECORD trial | 1 | 1 | 7 | 6 | 3 | **18** |
| VITAL | 0 | 1 | 7 | 5 | 3 | **16** |

†Adherence score calculated as per [22]. The 22 items were as follows: Title and abstract; Introduction (background and objectives); Methods (trial design, participants, interventions, outcomes, sample size, randomisation- sequence generation, allocation concealment- mechanism, implementation, blinding, statistical methods) Results (participant flow, baseline data, numbers analysed, outcomes and estimation, ancillary analyses, harms); Discussion (limitations, generalisability, interpretation).

**Supplementary Figure 1 All trials including *Golubic et al*.,**

When including the Golubic trial, there was suggestion of increased heterogeneity tau 0.1, I^2^ 12%, P=0.73


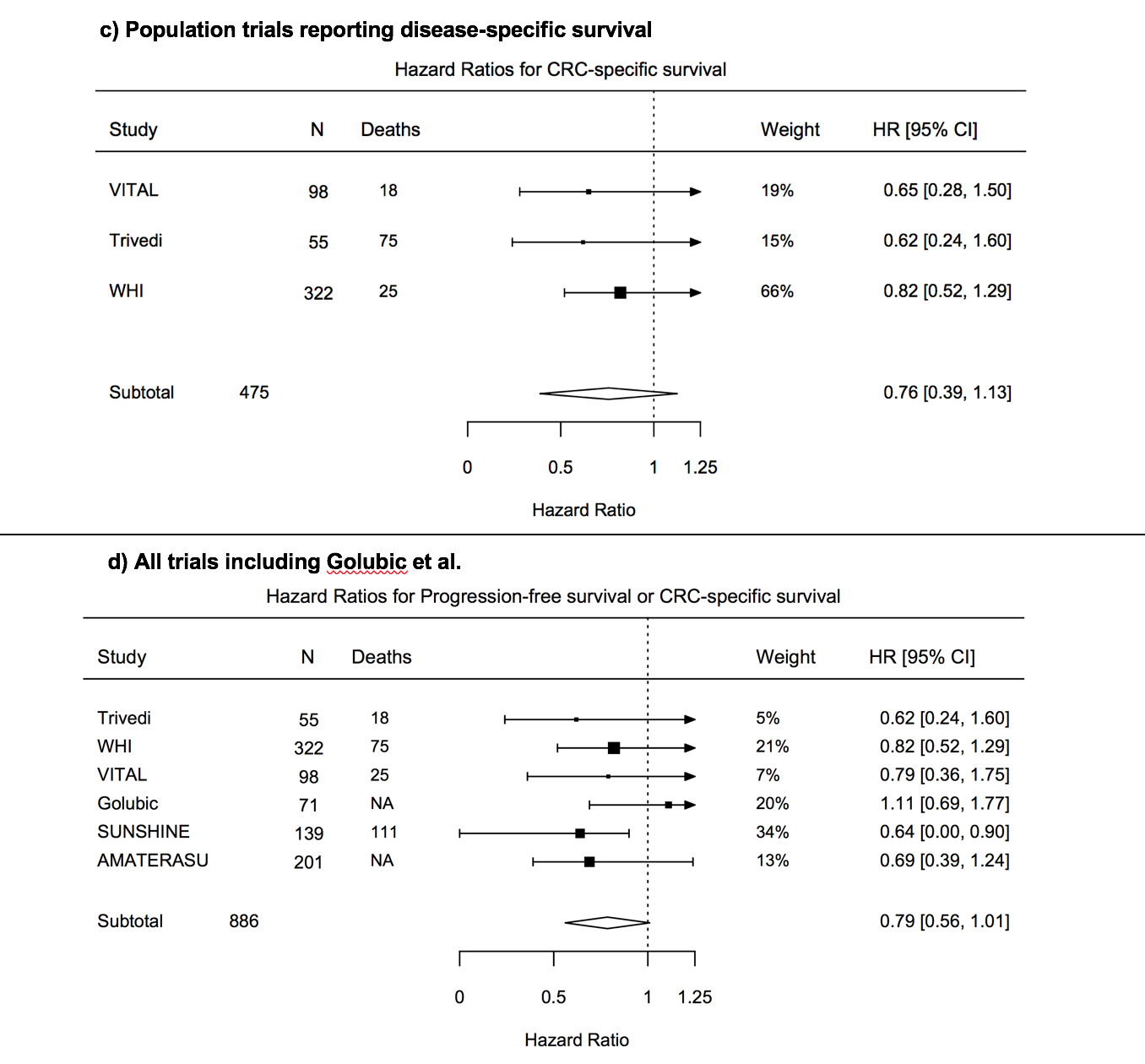


**Supplementary Figure 2 Funnel plot for studies included in overall meta-analysis**


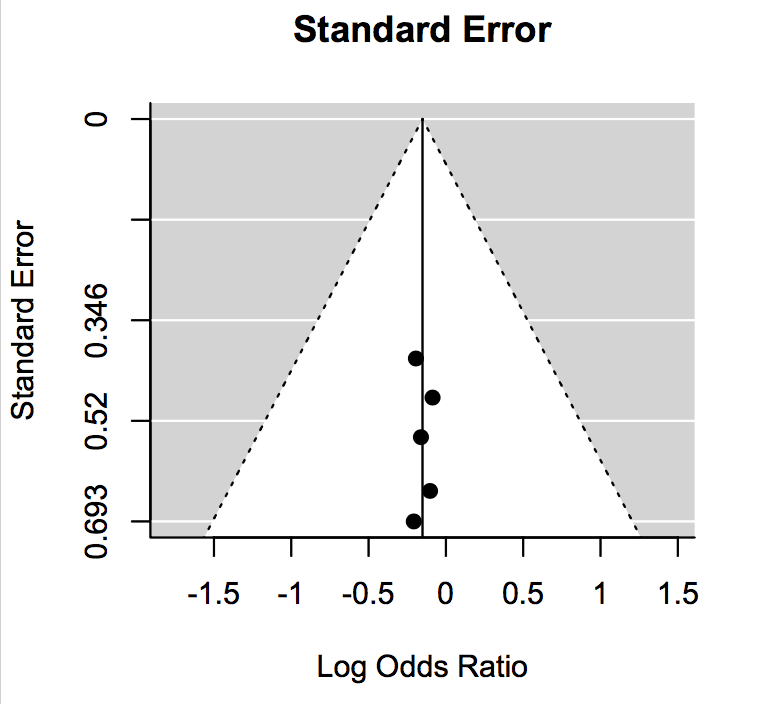


**Funnel plot for studies included in meta-analysis**

**Log HR**

HR used are for disease (CRC) specific survival for the Trivedi and Women’s Health initiative trials and progression free survival for the VITAL, SUNSHINE and AMATERASU trials. Egger’s test for funnel plot asymmetry t = -0.18, df = 3, p = 0.87
